# The complete mitochondrial genome sequence of *Chrysanthemum boreale* (Asteraceae)

**DOI:** 10.1080/23802359.2018.1468226

**Published:** 2018-04-27

**Authors:** So Youn Won, Jae-A Jung, Jung Sun Kim

**Affiliations:** a National Institute of Agricultural Sciences, Rural Development Administration, Jeonju, Republic of Korea;; b National Institute of Horticultural and Herbal Science, Rural Development Administration, Wanju, Republic of Korea

**Keywords:** Asteraceae, Chrysanthemum, mitochondrial genome, mitogenome, PacBio SMRT sequencing

## Abstract

Chrysanthemum is an important ornamental, herbal, and medicinal plant. We report the complete mitochondrial genome (mitogenome) sequence of *Chrysanthemum boreale*. The mitogenome is 211,002 bp in length, has a GC content of 45.36%, and contains 58 genes, including 35 protein-coding genes, three ribosomal RNA genes, and 20 transfer RNA genes. A phylogenetic analysis based on mitogenome protein sequences from various plants confirmed that *C. boreale* belongs to the Asteraceae family. This mitogenome will be useful in evolutionary and phylogenetic studies of *Chrysanthemum* and Asteraceae.

*Chrysanthemum,* a genus of flowering plants in the Asteraceae family, is famous for its diversity in flower shape, size, and colour and includes ornamental, commercial, and wild species. *Chrysanthemum boreale,* a wild species with small, yellow flowers, is distributed throughout eastern Asia (Kim et al. 2003) and has been used for medicinal and herbal purposes, due to its antibacterial, anti-inflammatory, and skin-regenerating properties (Kim et al. 2003; Kim et al. 2010; Kim et al. 2015). As *C. boreale* is resistant to white rust disease, this plant may be a suitable genetic resource to develop disease-resistant chrysanthemum cultivars (Park et al. 2014). Only two mitogenomes from the Asteraceae family (*Helianthus annuus* and *Diplostephium hartwegii*) have been deposited in GenBank and no mitogenome for the *Chrysanthemum* genus has been reported. In this study, we report the construction of the complete *C. boreale* mitogenome. This mitogenome will be useful in evolutionary and phylogenetic studies of *Chrysanthemum* and Asteraceae.

To construct the complex and dynamic mitogenome of *C. boreale* (GenBank BioSample SAMN07296937), long-read sequences were generated and assembled using PacBio’s Single Molecule Real-Time (SMRT) platform. The plant was collected from the Korea (N 35° 29′ 00″ E 126° 48′ 00″) and kept in National Institute of Horticultural and Herbal Science, Rural Development Administration with the ID of IT121002 (Hwang et al. 2013). A library was prepared from total genomic DNA using a SMRTbell Template Prep Kit 1.0 (Pacific Biosciences, PN 100-259-100) and sequenced on PacBio’s RS II platform using P6-C4 chemistry (DNAlink, Republic of Korea). The obtained raw reads were primarily assembled with the FALCON and FALCON-Unzip algorithms (Chin et al. 2016). Mitogenome-like sequences were searched by BLASTn against the complete mitogenome of *H. annuus* (GenBank Accessino number: CM007908.1), assembled into a single contig by CANU (Version 1.4), with the genome size set at 400,000, and finally circularized using MUMmer (Kurtz et al. 2004; Koren et al. 2017). The mitogenome was annotated using MITOFY and manually compared with other mitochondrial protein sequences (Alverson et al. 2010).

The *C. boreale* mitogenome was deposited in GenBank under accession number MH004292. The mitogenome is 211,002 bp in length, has a G+C content of 45.36%, and is predicted to contain 58 genes, including 35 protein-coding genes, three ribosomal RNA genes, and 20 transfer RNA genes. While most protein-coding genes had an ATG as the start codon, three (*atp6*, *ccmFn,* and *nad4L)* had an ACG, rendering them susceptible to C-to-U mRNA editing, which is common in plant mitochondria (Giegé and Brennicke 1999). Additionally, *mttB* had ATT as the start codon, which was commonly observed in *H. annuus* (Grassa et al. 2016). Three NADH dehydrogenase subunit genes, *nad1*, *nad2,* and *nad5,* are trans-spliced and seven genes (*nad4*, *nad7*, *cox2*, *ccmFc*, *rps1*, *rps3,* and *rps14*) contain introns, whereas the remaining 48 genes consist of a single exon.

Phylogenetic analysis was conducted using the mitogenomes of 12 plant species with *Arabidopsis thaliana* as an outgroup. The maximum likelihood (ML) tree based on the translated amino acid sequences of 20 common protein-coding genes (*atp1*, *atp4*, *atp8*, *atp9*, *ccmB*, *ccmC*, *ccmFn*, *cob*, *cox1*, *cox3*, *matR*, *mattB*, *nad3*, *nad4*, *nad4L*, *nad6*, *nad9*, *rpl5*, *rps4,* and *rps12*) showed that *C. boreale* and other Asteraceae species (*H. annuus* and *D. hartwegii*) form a monophyletic group supported by the bootstrap value of 100% (Figure 1). This complete mitogenome sequence of *C. boreale* is the first reported in the *Chrysanthemum* genus and represents a useful genetic resource to identify *C. boreale* and to conduct phylogenetic and evolutionary studies.

**Figure 1.**
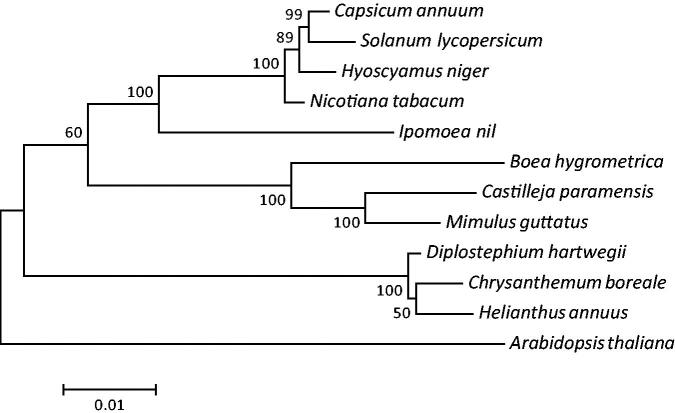
Maximum-likelihood phylogenetic tree based on the concatenated amino acid sequences of 20 protein-coding genes in 12 plant mitogenomes. *Arabidopsis thaliana* was used as an outgroup. Numbers at the nodes indicate bootstrap values for 1000 replicates. All the mitogenome sequences are available in GenBank: *Chrysanthemum boreale* (MH004292), *A. thaliana* (Y08501), *Boea hygrometrica* (NC_016741), *Capsicum annuum* (KJ865410), *Castilleja paramensis* (NC_031806), *Diplostephium hartwegii* (NC_034354), *Helianthus annuus* (NC_023337), *Hyoscyamus niger* (NC_026515), *Ipomoea nil* (NC_031158), *Mimulus guttatus* (NC_018041), *Nicotiana tabacum* (NC_006581), and *Solanum lycopersicum* (NC_035963).
